# Radiofrequency-induced thermotherapy of nasopharyngeal angiofibroma and immunohistochemical analysis of vessel proliferation: a case report

**DOI:** 10.1186/1752-1947-2-278

**Published:** 2008-08-16

**Authors:** Mira Krstulja, Milodar Kujundžić, Adelaida Halaj, Tamara Braut, Niko Cvjetković

**Affiliations:** 1 Department of Pathology, School of Medicine, University of Rijeka, Brace Branchetta, 51000, Rijeka, Croatia; 2 Clinic for Otolaryngology and Head and Neck Surgery, School of Medicine, University of Rijeka, Brace Branchetta, 51000, Rijeka, Croatia; 3 Clinical Department of Radiology, School of Medicine, University of Rijeka, Brace Branchetta, 51000, Rijeka, Croatia

## Abstract

**Introduction:**

Nasopharyngeal angiofibroma presents with symptoms of nasal obstruction and epistaxis. The treatment of choice is embolization followed by surgery.

**Case presentation:**

A 52-year-old man underwent surgery for nasopharyngeal angiofibroma after adjuvant radiofrequency-induced thermotherapy. To the best of the authors' knowledge, this is the first case of angiofibroma with clinical follow-up after thermocoagulation therapy supported by quantitative, double immunohistochemistry. We found this case of angiofibroma to be of interest owing to the presentation of symptoms leading to biopsy, the pathohistological observations obtained with synchronous Ki67/cluster of differentiation 34 and Ki67/smooth muscle actin immunohistochemistry and high pericyte proliferation.

**Conclusion:**

Coagulation of angiofibroma vessels followed by acquisition of a thick mantle of pericytes in a patient with a nasopharyngeal growth suggests that radiofrequency-induced thermotherapy could be a useful, palliative therapy for bleeding nasopharyngeal angiofibroma, supporting vessel maturation prior to surgical tumor removal.

## Introduction

Nasopharyngeal angiofibroma is considered to be a reactive, malformed, benign but aggressive neoplasm. Clinical staging and tumor embolization reduce surgical morbidity. The therapy protocol is influenced by hospital-related factors. Radiofrequency-induced thermotherapy (RFITT) is a minimally invasive surgical procedure that causes thermal ablation through coagulation and is used in the treatment of both head and neck diseases. We were unable to find reported cases of angiofibroma that were treated with RFITT, subjected to follow-up evaluation and had documented histological changes with time.

We present an unusual case of a 52-year-old man with nasopharyngeal angiofibroma that first appeared as a nasal polyp. Coagulation, thrombosis, sclerosis and pericyte proliferation occurred after RFITT. We looked for a change in angiofibroma cell proliferation through biopsies obtained before and after RFITT when the patient was free of bleeding episodes. The cell origin of vessel formation after thermocoagulation therapy was investigated. Our results are of interest for surgeons applying pre-operative thermal ablation therapy.

## Case presentation

**Figure 1:**
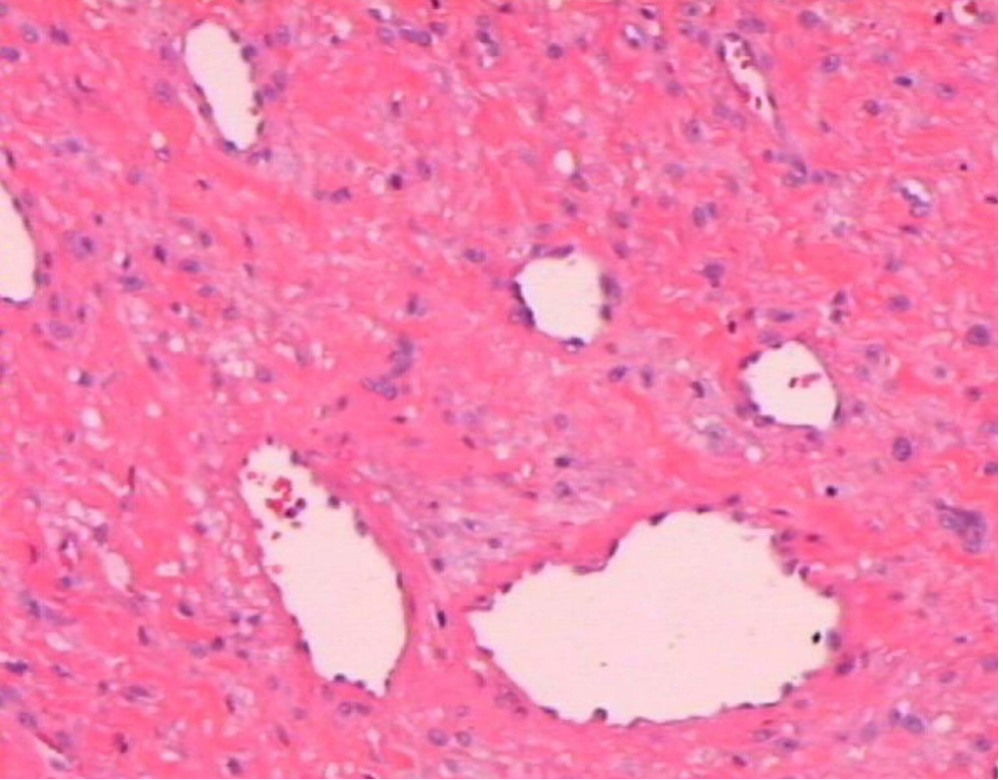
**Angiofibroma prior to radiofrequency-induced thermotherapy**. Hematoxylin and eosin stain, magnification ×10.

A 52-year-old white man, who experienced breathing difficulties and nasal speech for 15 months, was hospitalized for nasal polyps. A radiograph of his paranasal sinuses (21 January 2005) showed a soft tissue lesion in the mediosagittal line, suggesting a nasal polyp. A biopsy (18 February 2005) of the polyp revealed that it was immovable and provoked bleeding. The provided tissue (0.5 cm^3^) was diagnostic for nasopharyngeal angiofibroma after routine hematoxylin and eosin (H&E) staining (Figure 1), the stromal cells were negative for both cluster of differentiation (CD) 34 antigen and smooth muscle actin (SMA) antibodies and C-kit antibody was rarely detected in single cells.

**Figure 2:**
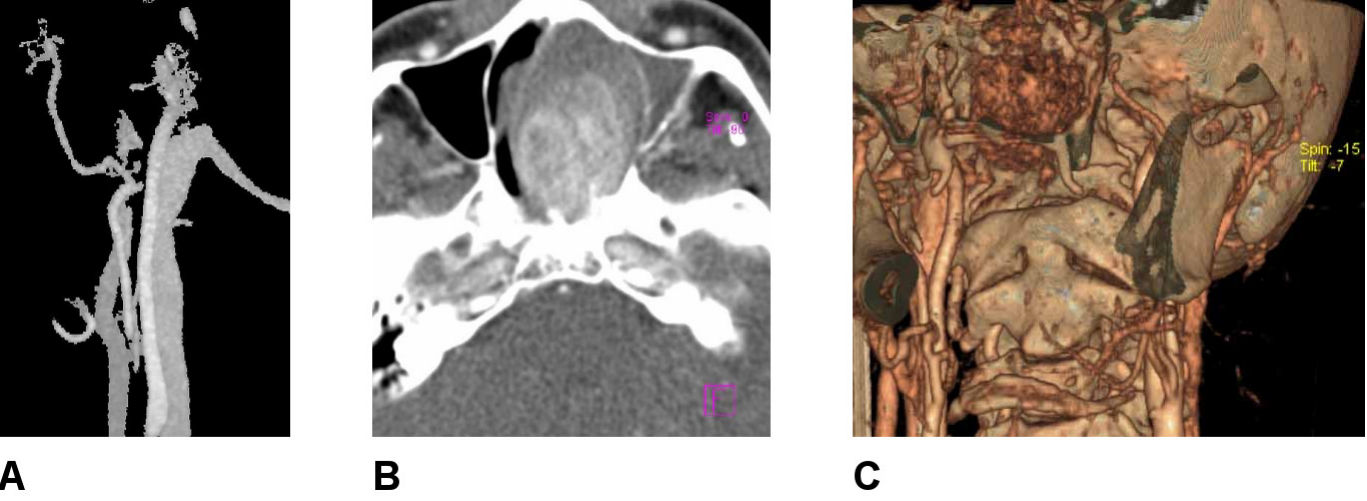
**Scans of a nasopharyngeal angiofibroma**. (A) Digital subtraction angiography (maximum intensity projection technique): the terminal branch of the left maxillary artery is at the hilus of the pathological angiofibroma neovascularization. (B) Computed tomography of the viscerocranium: nasopharyngeal angiofibroma seen with intravenous contrast. (C) The same tumor seen with computed tomography carotidography (volume rendering technique).

Digital subtraction angiography showed the pathological vascularization of the tumor (8 March 2005; Figure 2A). A computed tomography (CT) scan of the viscerocranium with intravenous contrast revealed a 56 mm × 48 mm large, soft tissue growth that filled the nasopharynx and extended to the left nasal cavity (24 February 2005; Figure 2B). A multiple slice CT carotidography (10 May 2005) revealed that there was blood supply to the tumor from the external carotid vessels (Figure 2C).

**Figure 3:**
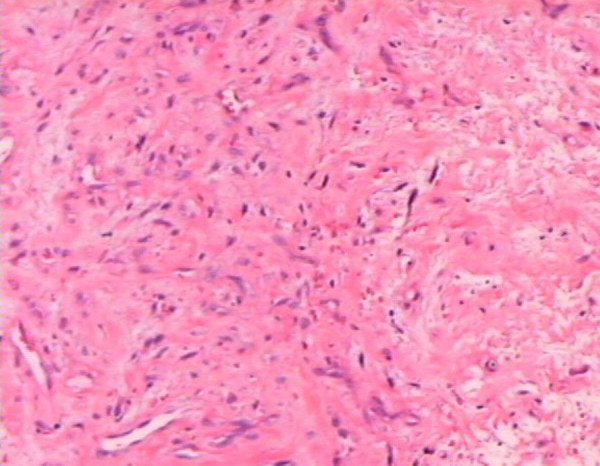
**Coagulation in angiofibroma (on the right), 3 weeks after radiofrequency-induced thermotherapy**. Hematoxylin and eosin stain, magnification ×10.

With a diagnosis of nasopharyngeal angiofibroma (Radkowski's stage Ib), the patient was subjected to RFITT using a Celon AG medical instrument (radiofrequency power, 15 to 20 W and a 5-minute application time). The therapy was performed three times over a 2-month period (1 June 2005, 9 June 2005 and 31 August 2005). The lesion did not bleed but hardened. The second surgical specimen (21 September 2005) was 5 cm^3 ^of angiofibroma tissue with multiple 2 to 3 mm centers of coagulation (Figure 3). After RFITT, the clinical symptoms were alleviated despite the incomplete reduction in tumor size. Staining for Ki67 showed low overall proliferation in the first biopsy but increased proliferation in the second (1% and 10%, respectively). A control CT scan (29 September 2005) of the epipharynx revealed a residual tumor, an enlarged left maxillary sinus with a missing medial wall, thickened mucosa without post-contrast opacification and no enlarged lymph nodes.

A third biopsy 10 months after RFITT provided 0.075 cm^3 ^of residual tumor with an overall Ki67 proliferation index of 10%. Plump SMA-positive and predominantly Ki67-negative cells were detached from the vessel wall and formed sheets resembling angiomyofibroblastoma after H&E staining. The second and third biopsies respected the recovery time from RFITT and were not complicated by hemorrhage.

One year after RFITT, angiography found no arteries feeding the residual tumor. The patient underwent surgery at another institution without prior embolization (no hypertrophic feeding arteries were found at repeated angiography before the operation).

**Figure 4:**
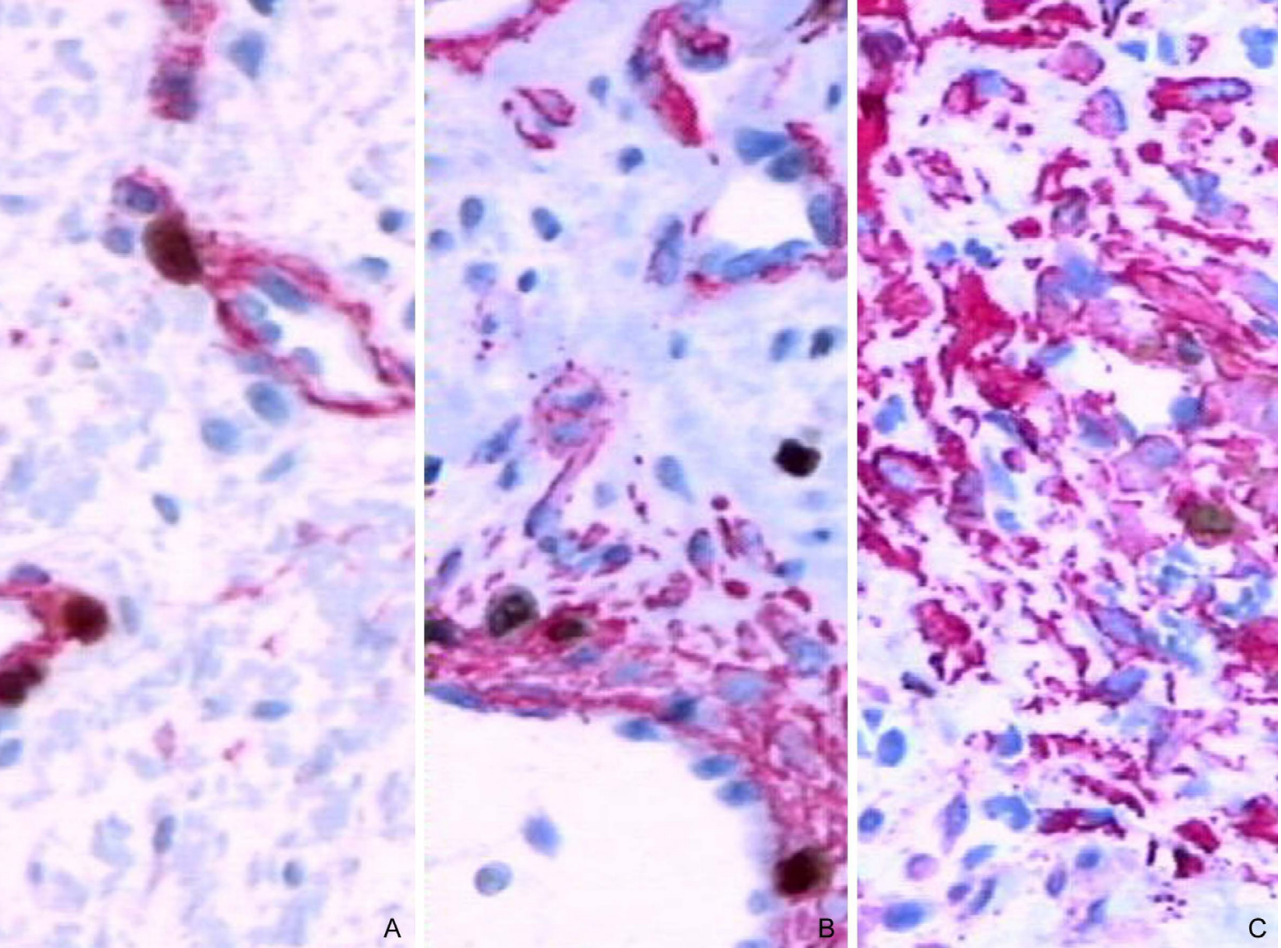
**Proliferation of pericytes in angiofibroma**. (A) Prior to radiofrequency-induced thermotherapy. (B) Three weeks after radiofrequency-induced thermotherapy. (C) Ten months after radiofrequency-induced thermotherapy, detachment of pericytes from the vessel wall. Magnification ×20. Ki67/SMA double immunohistochemistry. Ki67-positive nuclei of cycling cells were visualized using ChemMate DAB+ Chromogen. Cytoplasm of the endothelial cells and pericytes was visualized by fast red staining.

The primary intention was to reduce the tumor and alleviate the symptoms using RFITT before the operation. Double immunostaining was planned later because of increased Ki67 staining observed in the control biopsy after RFITT. Ki67 is a proliferation marker providing nuclear staining when the cell is in the S phase preparing to enter mitosis. To determine which cell type is proliferating in a tissue, a second differentiation marker is added, that is, CD34 for endothelial cells or SMA for pericytes. The immunohistochemical analysis of all three angiofibroma biopsies was repeated with a double-staining technique for both Ki67/CD34 and Ki67/SMA to distinguish between endothelial cell and pericyte proliferation over time (Figure 4A, B and 4C). Three parameters were used to quantify proliferation. The endothelial cell proliferation index (EPI) and pericyte proliferation index (PEPI) were defined as the percentage of Ki67-positive nuclei per 1000 cells for each cellular compartment. This was different from routine, less expensive single Ki67 immunostaining where the proliferation index takes into consideration all the cells in the tissue without distinguishing between vessel cells and stromal cells. The number of vessel sections per field was obtained and the results were expressed as microvessel density (MVD), which is the number of lumina per square millimeter. The proliferating capillary index (PCI) was defined as the percentage of vessel sections of any cell type whose nuclei stained positive for Ki67. The proliferation analysis results are shown in Table 1.

**Table 1:** Variables of cell proliferation and vessel proliferation in angiofibroma with time

Variable	Endothelial cell proliferation index (%)			Pericyte proliferation index (%)			Proliferating capillary index (%)			Microvessel density per mm^2^		
Order of biopsy	1	2	3	1	2	3	1	2	3	1	2	3
Mean*	8.34	11.18	9.10	16.04	19.36	20.59	38.5	42	54	138	180	226

1, 2 and 3: The first, second and third biopsies. *25 microscopic fields per variable (microscopic field 0.0415265 mm^2^).

Double immunohistochemical staining revealed higher proliferation indices for cells of the vessel compartment compared with single Ki67 staining of each routine biopsy. The EPI slightly decreased while the PEPI increased 10 months after RFITT. The third biopsy contained a large number of detached SMA-positive cells. There were scattered Ki67-positive nuclei of cells outside the vessel wall that were defined by neither CD34 nor SMA in all three biopsies. The MVD increased 20 days after RFITT and further increased with time. The PCI also increased with time. Measurements and images were obtained using a BX-40 Olympus microscope, Sony CCD-Iris color video camera and ISSA 3.1 software (Vamstec, Zagreb).

## Discussion

Nasopharyngeal angiofibroma is considered a malformation in juveniles [1-3], but does not exclude the unusual presentation of the disease in mature patients, as confirmed by this report and occasional reports from other authors [4]. While nasal polyps are not subjected routinely to CT or magnetic resonance imaging, these are established pre-operative diagnostic tools for nasopharyngeal angiofibroma.

The case presented here is of interest from both the clinical and the pathological points of view. The nasopharyngeal and sinonasal tracts are sites of different pathologies prone to epistaxis, such as the angiofibroma, angiectatic nasal polyp [5], and necrotizing angiocentric lesion. The stroma is different in these lesions and quite typical in angiofibroma. SMA decorates the stromal cells in certain nasal polyps. It is strongly positive in the vessel wall (pericytes) and occasionally in the stroma of angiofibromas [1,6], which may help in differential diagnosis.

In our case, two pathologies were present synchronously, a mucosal nasal polyp and an angiofibroma, making the diagnosis more complex as noticed by other authors [6]. The association between inflammatory nasal polyps and angiofibroma is not routinely expected, but once a biopsy is obtained, there are criteria to distinguish between nasal polyps arising through different pathogenic processes [7]. Nasopharyngeal angiofibroma is a rare event and biopsy is not advised. The first biopsy of our patient resulted from atypical extension of the tumor into the nasal cavity. The dates for the second and third biopsy were chosen with regards to the recovery period after RFITT. Although not a new disease, nasopharyngeal angiofibroma remains a clinical and scientific challenge. Thermocoagulation should be considered as a possible pre-operative protocol when embolization is not available.

The origin of angiofibroma is still under investigation. Zhang et al. [2] presented arguments for primary stromal change at the molecular level of angiofibroma organization. However, the origin of vessel formation is uncertain [8,9] and pericyte behavior in angiofibroma may be of interest. We were unable to find reports on pericyte proliferation in nasopharyngeal angiofibroma treated with RFITT. We find our observations of importance for the investigation of angiogenesis, angiofibroma and post-RFITT control biopsies. Our observations are in accordance with the purpose of the therapy, that is, to impede circulation and produce coagulation, thus reducing growth. The lesion was successfully treated surgically without pre-operative embolization, suggesting that RFITT might function as a pre-operative adjuvant therapy. Two years after RFITT, our patient is without symptoms or nasopharyngeal growth.

Histologically, both endothelial cell and pericyte proliferation were more accurately expressed with double immunohistochemistry compared with routine Ki67 staining. Pericyte proliferation was stronger than endothelial cell proliferation prior to therapy (PEPI 16.04%, EPI 8.34%). While the PEPI increased upon coagulation and progressed with time, the EPI did not. These results support the theory of angiofibroma as a maturing vasoformative lesion. Vessel formation is observed in inflammation, malformation, neovascularization of neoplasia and as a neoplastic event. Proliferation in vascular malformations has been studied previously [10,11]. Vessel formation in inflammation is diffuse except in granulomas. Malformations and neoplasias, including angiofibromas, behave as a 'body' in that they are fed and can be embolized, and angiofibromas are not considered neoplastic events. Malformations occurring with age are unusual but not unexpected. Zhang et al. [2] showed that angiofibroma stromal cells might be neoplastic. Our investigation of angiofibroma using double immunohistochemistry showed negligible proliferation outside the vascular compartment.

## Conclusion

We have presented a rare case of angiofibroma in a 52-year-old man with pericyte proliferation, supporting the maturation of the vessel compartment and revealing active angiogenic machinery (cooperation between endothelial cells and pericytes). We observed the divergent behavior of endothelial cells and pericytes after RFITT adjuvant therapy prior to surgery. Further studies of RFITT related to vessel behavior are needed. We found thrombosis and coagulation resulting from RFITT to function as equivalent to embolization prior to surgical therapy for angiofibroma. An analysis of vessel cell proliferation in tissues treated with thermal ablation might have broader clinical impact across medicine.

## Abbreviations

CD; Cluster of differentiation; CT: Computed tomography; EPI: Endothelial cell proliferation index; H&E: Hematoxylin and eosin; MVD: Microvessel density; PCI: Proliferating capillary index; PEPI: Pericyte proliferation index; RFITT: Radiofrequency-induced thermotherapy; SMA: Smooth muscle actin.

## Competing interests

The authors declare that they have no competing interests.

## Authors' contributions

MKr is the author of this study and performed the quantitative analysis of the double-stained immunohistological slides. MKu, TB and NC are surgeons who treated and observed the patient and provided the angiofibroma biopsy specimens. AH is our radiologist responsible for the acquisition of data and analysis and interpretation of data.

## Consent

Written informed consent was obtained from the patient for publication of this case report and any accompanying images. A copy of the written consent is available for review by the Editor-in-Chief of this journal.
